# Growth factor receptor plasticity drives therapeutic persistence of metastatic breast cancer

**DOI:** 10.1038/s41419-025-07591-3

**Published:** 2025-04-04

**Authors:** Mitchell Ayers, Marvis Monteiro, Aneesha Kulkarni, Julie W. Reeser, Emily Dykhuizen, Sameek Roychowdhury, Michael K. Wendt

**Affiliations:** 1 Department of Medicinal Chemistry and Molecular Pharmacology, Purdue University, West Lafayette, IN 47907 USA; 2 Purdue Institute for Cancer Research, Purdue University, West Lafayette, IN 47907 USA; 3 Comprehensive Cancer Center and James Cancer Hospital, The Ohio State University, Columbus, OH 43210 USA; 4 Department of Internal Medicine, Division of Medical Oncology, The Ohio State University, Columbus, OH 43210 USA; 5 Department of Internal Medicine, University of Iowa, Iowa City, IA 52242 USA; 6 Holden Comprehensive Cancer Center, University of Iowa, Iowa City, IA 52242 USA

**Keywords:** Breast cancer, Receptor pharmacology, Breast cancer, Drug development

## Abstract

Metastatic breast cancer (MBC) remains a therapeutic challenge due to the persistence of minimal residual disease (MRD) and tumor recurrence. Herein we utilize a model of MBC that is sensitive to inhibition of fibroblast growth factor receptor (FGFR), resulting in robust regression of pulmonary lesions upon treatment with the FGFR inhibitor pemigatinib. Assessment of the remaining MRD revealed upregulation of platelet-derived growth factor receptor (PDGFR). Functionally, we demonstrate increased response to PDGF ligand stimulation following pemigatinib treatment. Depletion of PDGFR did not alter tumor growth under control conditions but did delay tumor recurrence following a treatment window of pemigatinib. To overcome this therapeutic hurdle, we found that inhibition of DNA methyltransferase 1 (DNMT1) prevents pemigatinib-induced cellular plasticity. Combined targeting of FGFR and DNMT1 prevented induction of PDGFR, enhanced pulmonary tumor regression, slowed tumor recurrence, and prolonged survival. These findings enhance our understanding of cellular plasticity during states of treatment-induced MRD and suggest that inhibition of DNA methylation could augment current approaches being used to treat MBC.

## Introduction

Breast cancer (BC) is the most diagnosed cancer and second-leading cause of cancer-related mortality among U.S. women. The 5-year survival rate for BC patients diagnosed during the early stages is nearly 100%, while only 31% of patients with metastatic disease survive five years [1]. This highlights the need for improved therapeutic strategies that focus specifically on metastatic tumor biology. As opposed to some cancer types that remain dependent on a single pathway, acquired and intrinsic drug resistance poses a substantial challenge to targeted therapies aimed at treating metastatic breast cancer (MBC). A consistent explanation of drug resistance in MBC lies in the enhanced ability of these tumors to transition between different cell states and growth programs upon application of therapeutic stress [2].

Many factors contribute to cell state changes during metastasis and therapeutic resistance, including epithelial-to-mesenchymal transition (EMT), cellular dormancy, and epigenetic alterations. EMT is a process by which epithelial-derived carcinoma cells undergo gene expression changes that can manifest as subtle to profound state changes, broadly referred to as mesenchymal [3]. The fibroblast growth factor receptor (FGFR) signaling pathway is upregulated during EMT, and these molecular events are associated with an increased incidence of metastatic disease [4]. This makes FGFR an appealing therapeutic target, especially since several FGFR inhibitors are approved by the U.S. Food and Drug Administration for the treatment of other cancers in which the receptor becomes activated by genetic mutation. However, activation of FGFR signaling in BC also fuels cellular plasticity, supporting epigenetic events that allow cells to overcome stresses of the metastatic process [5, 6]. As such, targeting FGFR in MBC has not made a major clinical impact.

Concurrent with the execution of EMT is the entry of cancer cells into drug-persistent states of dormancy. Due to decreased proliferation and increased integration into the normal tissues they inhabit, these minimal residual disease (MRD) populations are highly resistant to anti-cancer therapeutics [7]. Factors that allow for persistence of MRD during drug treatment and re-emergence of disease following cessation of therapy are only beginning to be understood. In particular, whether distinct survival factors are upregulated in response to specific therapeutics remains to be defined. This epigenetic reprogramming can include modifications to DNA, histones, and other post-transcriptional, post-translational events. Overall, these epigenetic events contribute to cancer cell plasticity, establishment of MRD, and acquired therapeutic resistance [8, 9].

Here, we investigated the mechanistic underpinnings of cellular persistence during drug-induced MRD. To do this we utilized the 4T07 syngeneic model of triple-negative breast cancer (TNBC). Treatment of this model with the FGFR inhibitor pemigatinib resulted in a substantial regression of pulmonary lesions but failed to fully eradicate disease. We hypothesized that characterization of this MRD would yield actionable events that could be targeted to further reduce disease burden and delay tumor recurrence. We demonstrated that disease persistence in response to FGFR blockade is supported by a dynamic activation of platelet-derived growth factor receptor (PDGFR) signaling. As a means of limiting cellular plasticity, we used a DNA methylation inhibitor to prevent PDGFR upregulation and reduce survival of MRD. Overall, our studies demonstrate an actionable mechanism of MRD persistence.

## Results

### Pemigatinib inhibits pulmonary tumors and alters the tumor microenvironment

**Fig. 1:**
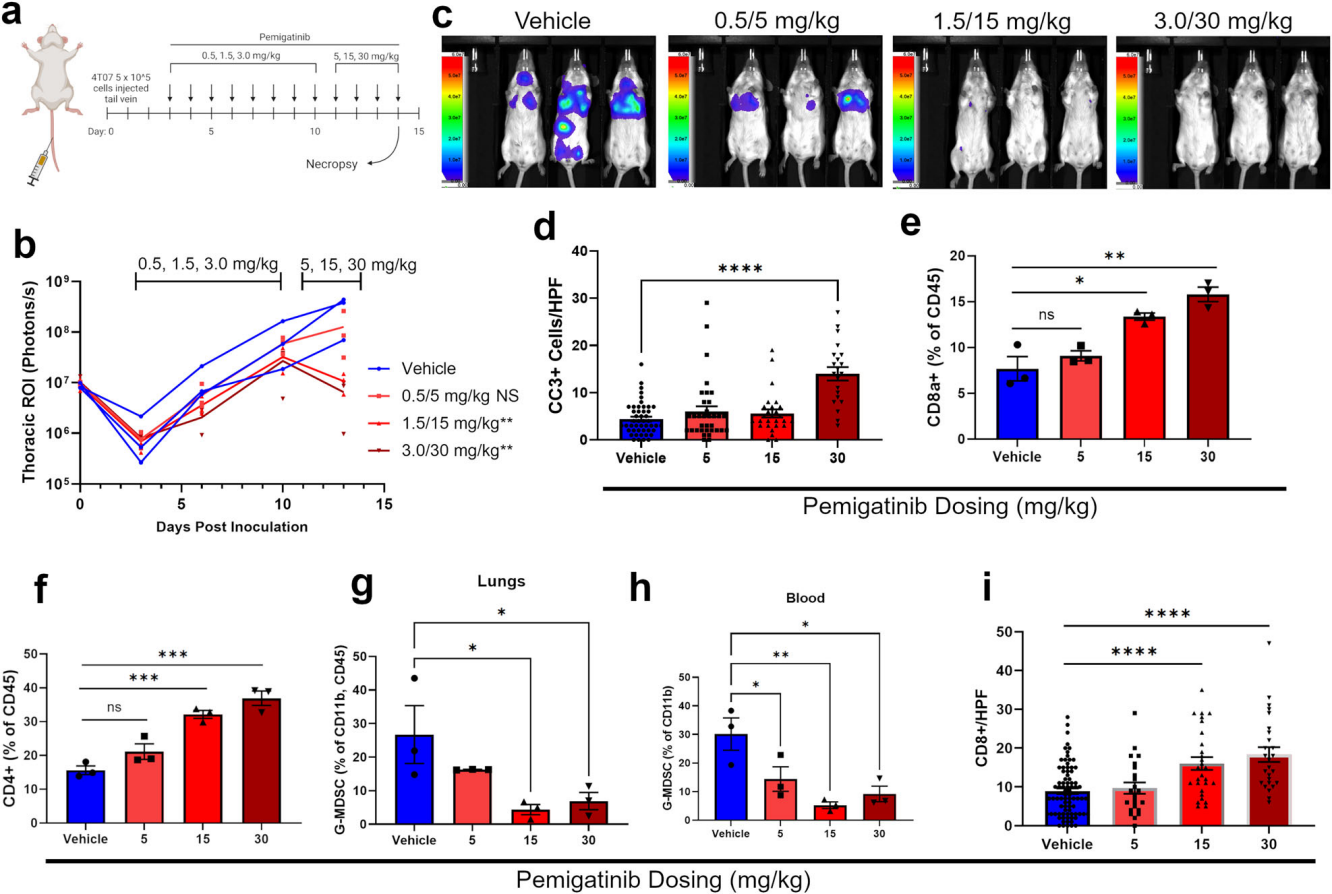
Pemigatinib induces robust increase in T-cells in pulmonary breast cancer (BC) tumors. **a** Study timeline schematic representing a tail vein inoculation of 4T07 (5 × 10^5^ cells) into approximately 6-week-old female BALB/c mice. On day 3 mice were randomized and pemigatinib was initially dosed at 0.5, 1.5, and 3.0 mg/kg by oral gavage daily then escalated to 5, 15, and 30 mg/kg. **b** Bioluminescent imaging was tracked over time of 4T07 firefly-positive tumors. The graph represents the pulmonary region of interest (ROI) from indicated study days with 3 mice per group. **c** Bioluminescent images from day 13 prior to necropsy. **d** After their last pemigatinib dose on day 14, mice were necropsied, then lungs were sectioned for IHC and stained for cleaved caspase 3 (CC3). The number of CC3+ cells were counted in several high-powered fields across three biological replicates. **e**–**h** Lungs dissociated into single cells or whole blood were used for flow cytometric analysis. Data represent 3 biological replicates. **e** CD8a+ T-cells as a percentage of CD45+ cells are represented for each pemigatinib dose group. **f** CD4 + T-cells as a percentage of CD45+ cells are represented. **g** The percentage of G-MDSCs (Ly6C–, Ly6G + ) in the CD11b+ and CD45+ populations is represented from dissociated lungs. **h** Whole blood collected via cardiac puncture from pemigatinib treated mice were analyzed by flow cytometry, and the graph represents the G-MDSCs (Ly6C–, Ly6G + ) as a percentage of the CD11b+ population. **i** CD8a+ cells were counted in IHC sections per high-powered field as in panel d. All calculated *P*-values were done using a two-tailed unpaired Student’s *t-*test. Data are the mean +/− s.e.m.

**Fig. 2:**
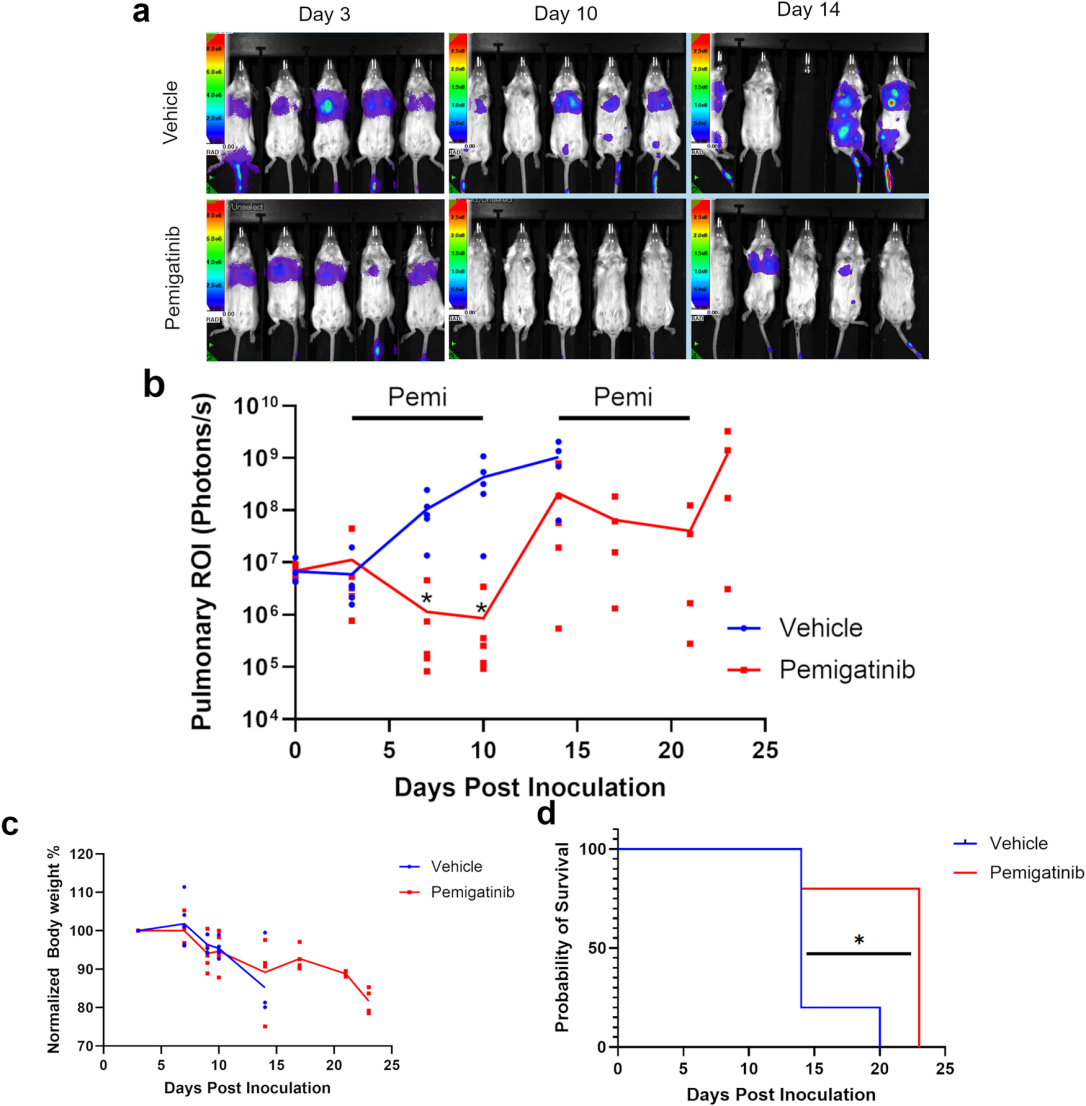
Pemigatinib prolongs survival of pulmonary tumor bearing mice. **a** 4T07 cells (5 × 10^5^) were injected into the tail vein of approximately 6-week-old female BALB/c mice, and after 3 days mice were randomized prior to dosing of pemigatinib at (25 mg/kg). BLI of vehicle vs pemigatinib-treated mice on days 10 and 14. **b** Pulmonary ROI values from BLI were graphed on a log scale over time for each group. *Indicates *P* < 0.05 between groups at the indicated time points as determined by an unpaired Student’s *t*-test. Mice were treated with second round of pemigatinib (10 mg/kg) as indicated. The trend lines indicate the mean value of each over time. **c** Normalized body weights (day 3 set to 100%) for the two treatment groups. **d** Kaplan-Meier plot comparing pemigatinib treatment and vehicle groups (*n* = 5 mice per group). Data were analyzed by a log-rank test.

Our previous studies established the ability of an experimental inhibitor of FGFR to delay the growth of the 4T07 BC model following delivery of these cells to the lungs via tail vein inoculation [10]. Therefore, we evaluated the efficacy of pemigatinib, a U.S. Food and Drug Administration–approved FGFR inhibitor, in this model of disseminated BC (Fig. 1a). Using a dose escalation approach in combination with longitudinal bioluminescent imaging (BLI), we were able to establish 15 mg/kg as an effective dose capable of reducing the pulmonary tumor burden (Fig. 1b, c). In addition to decreasing total tumor burden, we also observed increased levels of apoptosis via histological staining for cleaved caspase 3 (CC3) (Fig. 1d and Supplementary Fig. 1a). Our previous studies also indicate changes in the pulmonary tumor immune microenvironment upon systemic inhibition of FGFR [10]. Consistent with these data, we found that treatment with effective concentrations of pemigatinib resulted in increased numbers of T-cells and decreased numbers of myeloid-derived suppressor cells as assessed by flow cytometry and immunohistochemistry (IHC) (Fig. 1e–i, Supplementary Fig. 1a). Following our dose-finding studies in Fig. 1, treatment with pemigatinib at a fixed dose significantly reduced pulmonary tumor burden in these mice (Fig. 2a, b). In fact, pemigatinib treatment caused pulmonary tumor regression over a 7-day course of treatment (Fig. 2b). However, after the pemigatinib treatment window, tumors rapidly rebounded, growing at a faster rate than control tumors following the original inoculation (Fig. 2b). A second dosing regimen of pemigatinib again decreased pulmonary tumor burden (Fig. 2b). Overall, pemigatinib treatments sustained body condition and increased median survival from 14 to 23 days (Fig. 2c, d). These findings demonstrate that pemigatinib alters the tumor immune microenvironment and leads to robust, but incomplete, regression of tumors growing in the lung, a common site of BC metastasis.

### PDGFR expression is regulated by FGFR signaling

**Fig. 3:**
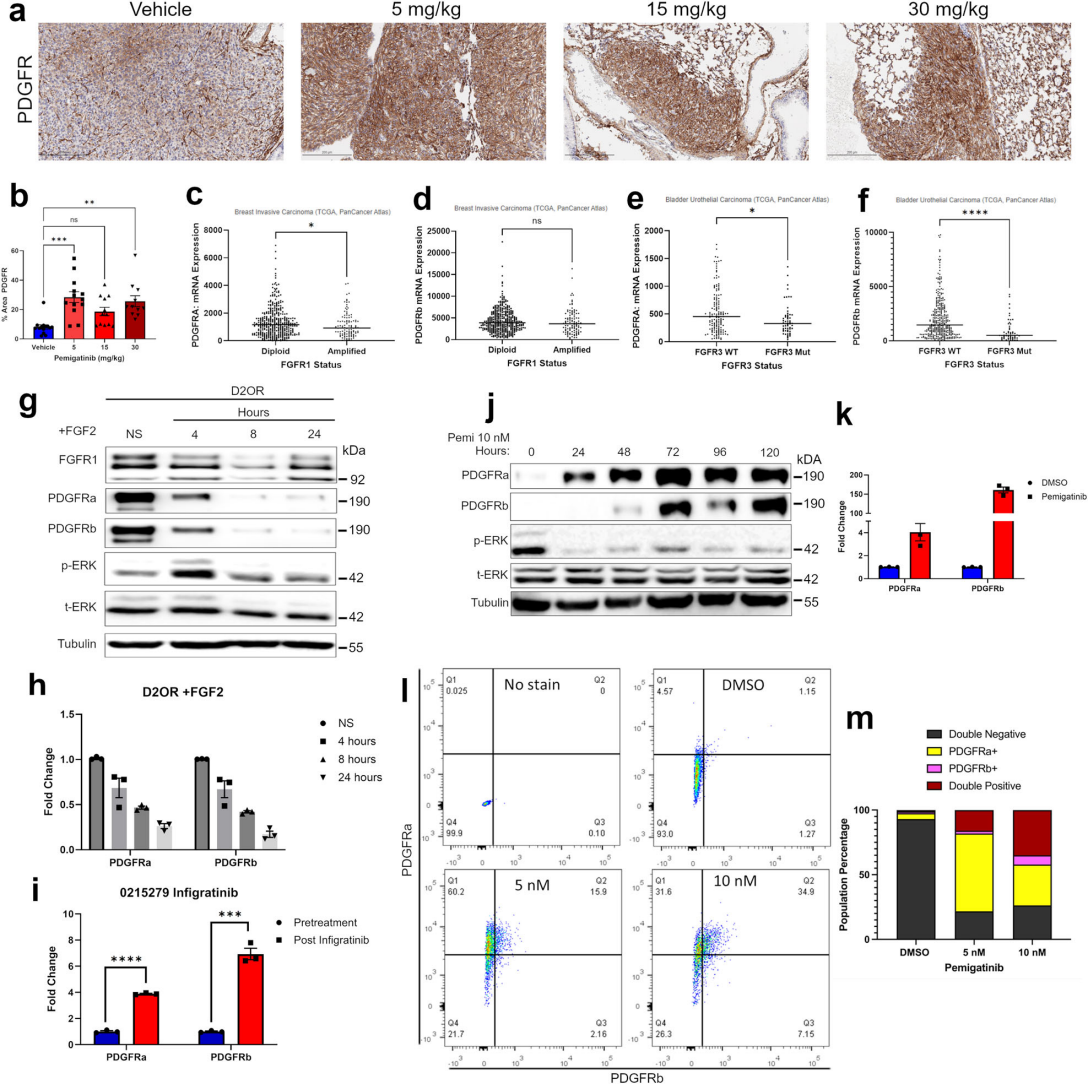
PDGFR expression is regulated by FGFR signaling. **a** Staining for PDGFR was performed on IHC sections from the in vivo experiments described in Fig. 1. **b** The percentage area of PDGFR-positive staining for several high-powered fields was calculated using image J across 3 biological replicates. **c**, **d** Data comparing PDGFRα (c) and PDGFRβ (d) in the Breast Invasive Carcinoma PanCancer atlas data set based on their FGFR1 status (diploid vs amplified). **e**, **f** Data comparing PDGFRα (e) and PDGFRβ (f) in the Bladder Urothelial Carcinoma PanCancer atlas data set based on their FGFR3 mutational status. Data are compared using a two-tailed unpaired Student’s *t-*test where * = *P* < 0.05 and **** = *P* < 0.0001. **g** D2.OR cells were stimulated with FGF2 (20 ng/µL) for the indicated amounts of time. Following stimulation cells were lysed and analyzed by immunoblot for expression of FGFR1, PDGFR (α and β), ERK1/2 (phospho and total), and tubulin. **h** mRNA fold change as measured by RT-PCR was quantified in D2.OR lines after the indicated number of hours of FGF2 stimulation. Data represents the mean of 3 biological replicates. **i** RT-PCR data for PDGFR (a and b) from an MBC patient carrying an *FGFR1* amplification prior to and after infigratinib dosing regimen of 1 week. Data represents 3 technical replicates of PCR completed on single RNA isolations from each condition. **j** 4T07 cells were treated with pemigatinib (10 nM) for the indicated amounts of time. Cells were lysed and analyzed by immunoblot for expression of PDGFR (α and β), ERK1/2 (phospho and total), and tubulin. **k** 4T07 cells were treated with pemigatinib (10 nM) for 24 h and cells were analyzed by RT-PCR for expression of PDGFRa and PDGFRb. Data represent the mean of 3 biological replicates. **l–n** 4T07 cells treated in vitro with 5 or 10 nM pemigatinib for 72 h then prepared for flow cytometry. **l** Dot plot of flow cytometric analyses of PDGFRα vs PDGFRβ. **m** Populations portions for expression of PDGFRα and PDGFRβ from flow cytometry data were graphed as parts of a whole.

Given the rapid recurrence of pulmonary disease following pemigatinib treatment, we sought to understand mechanisms of persistence that allowed survival of the MRD. Through our assessment of changes in the tumor microenvironment upon pemigatinib treatment, we stained histological sections for markers of cancer-associated fibroblasts, including PDGFR. This approach revealed a significant increase in tumor cell expression of PDGFR upon treatment with pemigatinib (Fig. 3a, b). Complementary to these findings, analysis of TCGA data revealed that both FGFR1-amplified BCs and bladder cancers harboring an FGFR3-activating mutation expressed less PDGFR as compared to their wild-type counterparts (Fig. 3c–f). These data suggest that aberrant FGFR signaling could be downregulating PDGFR expression. To directly address this question, we utilized the D2.OR cell line, an indolent model that we have recently shown can be driven by FGF2 stimulation [11, 12]. These cells express high levels of FGFR1 and PDGFR (Fig. 3g). Stimulation of these cells with FGF2 decreased both mRNA and protein levels of PDGFRα and PDGFRβ (Fig. 3h). Consistent with our model systems, analysis of sequential tumor biopsies from an FGFR1-amplified, triple-negative, MBC patient before and after 1 week of treatment with the FGFR inhibitor infigratinib similarly indicated upregulation of PDGFR (Fig. 3I). Returning to the 4T07 model, we observed in vitro treatment with pemigatinib induced PDGFR expression in a time frame that preceded cell doubling (Fig. 3j, k). Additionally, use of flow cytometry indicated that there is not a pre-existing PDGFR^high^ subpopulation that is selected for with pemigatinib treatment, but instead drug treatment shifted 4T07 cells to a PDGFRα single-positive, then to a PDGFRα/PDGFRβ double-positive phenotype (Fig. 3l, m). These data clearly demonstrate a reciprocal and dynamic regulation of PDGFR expression as a downstream output of FGFR signaling.

### FGFR blockade primes response to PDGF ligand stimulation

**Fig. 4:**
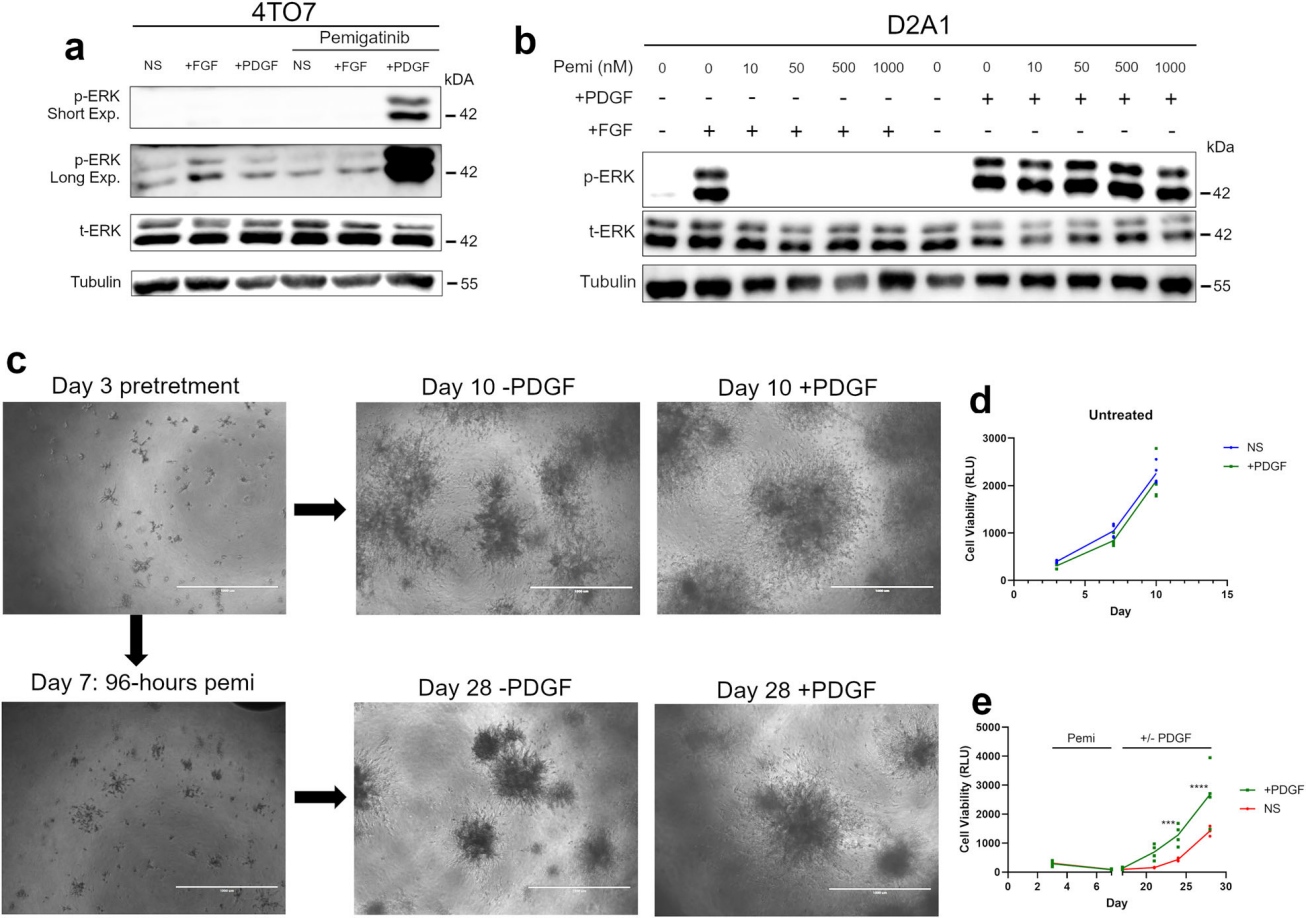
FGFR blockade primes response to PDGF ligand stimulation. **a** 4T07 cells were left untreated or treated with pemigatinib for 14 days. Following this pretreatment, cells were stimulated with FGF2 (20 ng/µL) or PDGF-bb (100 ng/µL) for 5 min. Cells were lysed and analyzed by immunoblot for ERK1/2 (phospho and total) and tubulin. **b** D2.A1 cells were pretreated with the indicated concentrations of pemigatinib for 24 h and then stimulated with either FGF2 (20 ng/µL) or PDGF-bb (100 ng/µL) for 5 min. Cells were similarly analyzed as in panel a. **c** 4T07 cells (2000 cells) were seeded under 3D culture conditions for 3 days at which point the cultures were left untreated (top row) or treated with pemigatinib (50 nM; bottom row) for 96 h. Following drug treatment, cultures were allowed to recover in the absence or presence of PDGF-bb (100 ng/µL). Representative brightfield images are shown at the indicated days and treatment conditions. **d** 3D cell growth of untreated 4T07 cells in the absence or presence of exogenous PDGF. Data are representative of 3 biological replicates. **e** 3D cell growth of pemigatinib-treated 4T07 cells followed by treatment with PDGF. Data are 4 technical replicates, representative of 3 biological replicates. Data are the mean +/− s.e.m. where all calculated *P*-values were done using a two-tailed unpaired Student’s *t-*test.

We next investigated the functional significance of PDGFR upregulation in facilitating differential response and resistance to pemigatinib. Stimulation with PDGF ligand only induced downstream phosphorylation of Erk1/2 after 4T07 cells were treated with pemigatinib (Fig. 4a). In contrast to the 4T07 cells, the metastatic D2.A1 cell line is not sensitive to pemigatinib. Stimulation of these cells with PDGF causes Erk1/2 phosphorylation irrespective of pemigatinib (Fig. 4b). Pretreatment of these cells with pemigatinib blocked signaling through FGFR; however, PDGF-mediated phosphorylation of Erk1/2 was not affected (Fig. 4b). We then developed a bioluminescent, 3D culture method to assess both response and recurrence of 4T07 cells to pemigatinib. The 4T07 cells expressing firefly luciferase were plated in a basement membrane extract matrix, and the cells were allowed to establish themselves for 3 days (Fig. 4c). At this point, the cultures were treated with pemigatinib for 96 h at a concentration that led to near background luminescence readings, recapitulating the MRD we observed in vivo (Fig. 4c–e). After this treatment period, the drug was washed off and the cells were allowed to recover in the presence or absence of exogenous PDGF ligand. Consistent with our signaling assays, PDGF stimulation had no effect on growth of control 4T07 cells (Fig. 4c; top row, d). In contrast, supplementation with exogenous PDGF significantly increased cellular recovery from pemigatinib-induced experimental MRD (Fig. 4c; bottom row, e). Together, these data suggest that inhibition of FGFR kinase activity increases protein expression of functional PDGFR and is capable of facilitating cellular recovery following induction of MRD.

### PDGFR is transiently upregulated following pemigatinib treatment

**Fig. 5:**
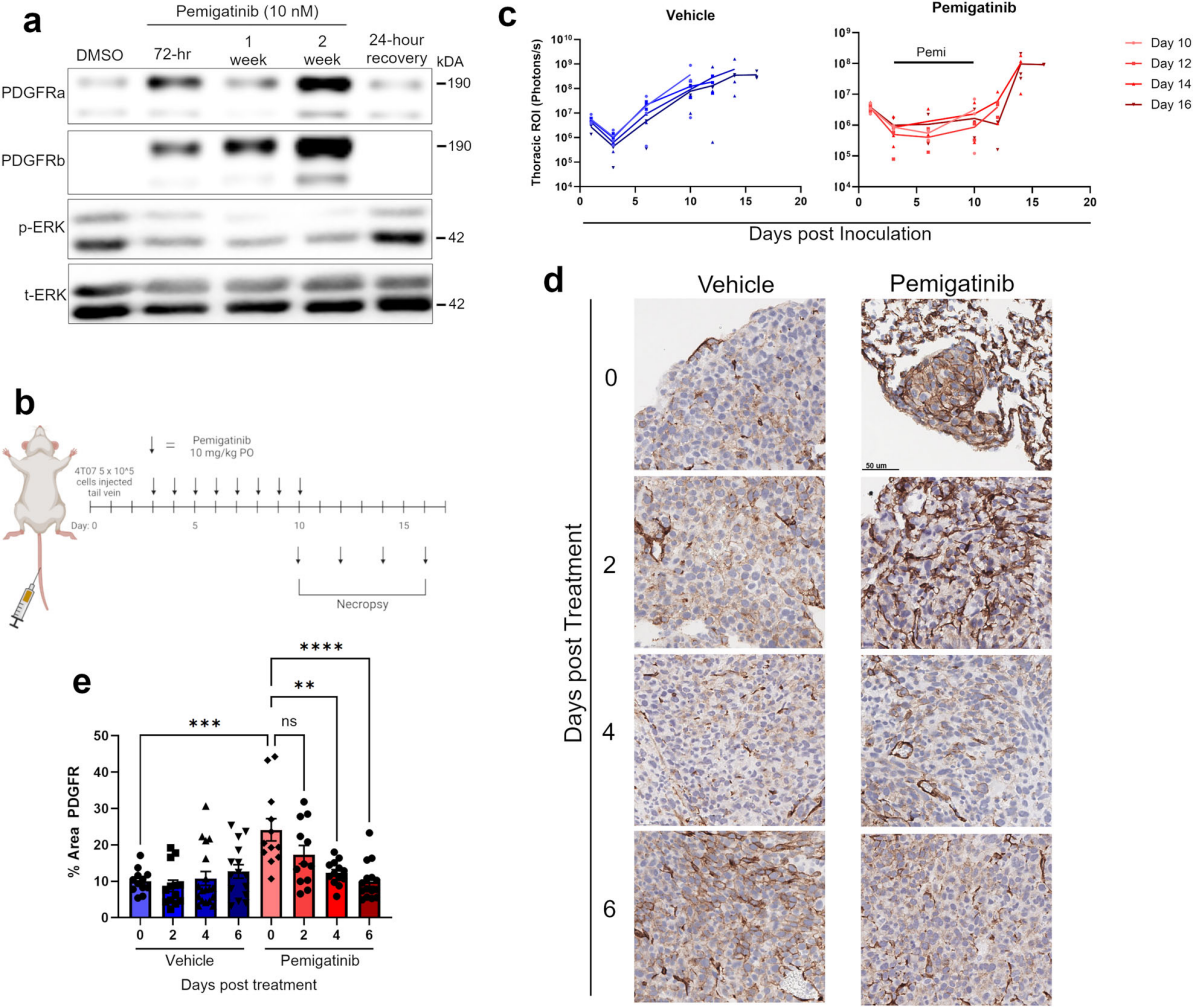
PDGFR is transiently upregulated following pemigatinib treatment. **a** 4T07 cells were treated with a time course of pemigatinib (10 nM) for 2 weeks at which point cells were allowed to recover for 24 h. Cells were lysed and analyzed by immunoblot for expression of PDGFR (α and β), and ERK1/2 (phospho and total) at the indicated time points. **b** Schematic of in vivo experiment timeline. 4T07 (5 × 10^5^) cells were inoculated into the tail vein of approximately 6-week-old female BALB/c mice. On day 3, mice were randomized into vehicle or pemigatinib treatment groups and began daily oral gavage dosing until day 10. Starting on day 10, 3 mice from either vehicle or pemigatinib group were necropsied and their lungs were fixed and prepared for IHC. **c** Pulmonary ROIs over time derived from the bioluminescent images were graphed over time with 3 mice per time point in each group. **d** IHC sections of PDGFR staining from vehicle and pemigatinib treatment groups at the indicated number of days post-treatment. **e** PDGFR IHC was quantified as a percent area of PDGFR positivity in identified tumor using imageJ. Data points are individual high-powered fields taken across each of the 3 biological replicates for the indicated time points. All calculated *P*-values were done using a two-tailed unpaired Student’s *t-*test. Data are the mean +/− s.e.m.

Many mechanisms of drug resistance result from selection events leading to changes in oncogenic drivers that are retained even after the selective pressure of an inhibitor. To establish whether the PDGFR upregulation induced by pemigatinib was sustained or transient in nature, we conducted an in vitro time course of pemigatinib treatment. This approach revealed upregulation of PDGFRα and PDGFRβ concurrent with diminution of the constitutive Erk1/2 phosphorylation that characterizes the 4T07 cell model (Fig. 5a). However, PDGFR expression returned to baseline levels within 24 h of pemigatinib cessation. To extend these findings to our in vivo system, mice bearing 4T07 pulmonary tumors were treated with pemigatinib to induce MRD (Fig. 5b, c). After this treatment, mice were necropsied at increasing time points and tumors were analyzed by flow cytometry and histology. As before, we found that pemigatinib significantly increased PDGFR expression compared to control (Fig. 5d, e). Vehicle-treated, tumor-bearing mice did not demonstrate any significant changes in PDGFR expression through tumor progression (Fig. 5e). Importantly, four days after pemigatinib treatment was stopped, PDGFR protein expression returned to control levels (Fig. 5e). Flow cytometry indicated that changes in T-cell recruitment with pemigatinib were also transient in nature, whereas decreased myeloid-derived suppressor cell (MDSC) mobilization did not normalize until 6 days after cessation of treatment (Supplementary Fig. 2). These findings indicate that growth factor receptor plasticity and modulation of the tumor immune microenvironment induced by FGFR inhibition are transient changes that take place during MRD but normalize upon treatment withdrawal.

### Pulmonary fibroblasts support cancer cell survival during pemigatinib-induced MRD

**Fig. 6:**
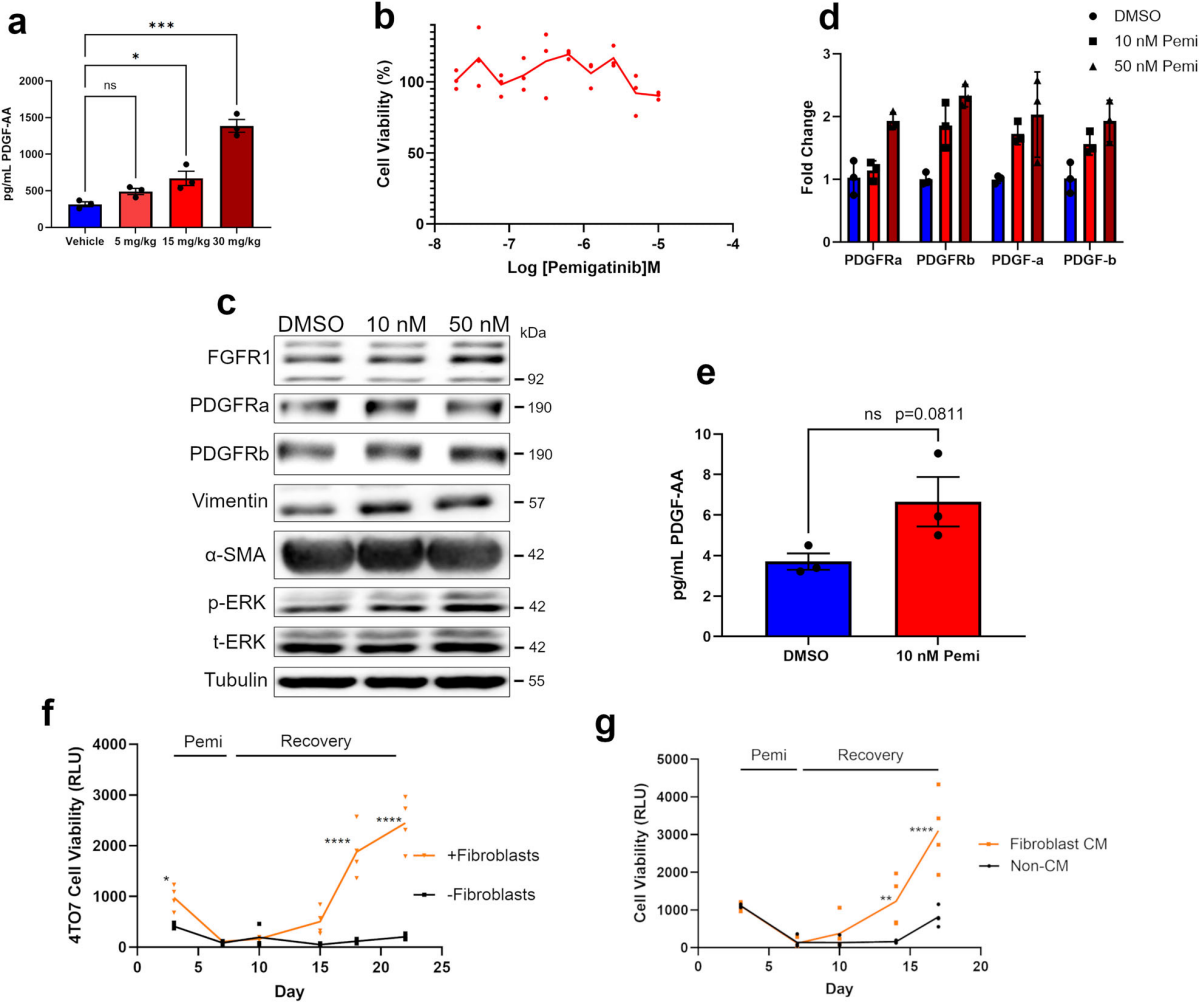
Pulmonary fibroblasts support cancer cell survival during pemigatinib-induced MRD. **a** Blood plasma was collected from mice treated with pemigatinib described in Fig. 1. PDGF-AA was detected by ELISA. Data are three technical replicates from the pooled samples of 3 mice per condition. **b** Pulmonary fibroblasts were isolated from tumor-naïve BALB/c mice, and cell viability was assessed following 4 days of treatment with the indicated concentrations of pemigatinib. Data are 3 biological replicates. **c** Pulmonary fibroblasts were treated with the indicated concentrations of pemigatinib for 72 h, and cells were lysed and analyzed by immunoblot expression of FGFR1, PDGFRα, PDGFRβ, vimentin, α-SMA, ERK1/2 (phospho & total), and tubulin. **d** Pulmonary fibroblasts were treated as in panel c and analyzed by RT-PCR for expression of PDGFRa, PDGFRb, PDGF-a, and PDGF-b. Data represents 3 technical replicates. **e** PDGF-AA was quantified by ELISA from conditioned media of pulmonary fibroblasts treated with DMSO vs. pemigatinib for 72 h in serum-free media. Data are 3 technical replicates of single ex-vivo culture. **f** Co-culture of 4T07 and pulmonary fibroblasts at a 1:10 ratio were grown under 3D culture conditions. Cells were treated with pemigatinib from days 3–7 post-plating and then allowed to recover. Longitudinal viability of the 4T07 cells was tracked overtime using luciferin. Data are 3 biological replicates. **g** 4T07 cells were grown under 3D culture conditions. Cells were treated with pemigatnib from day 3 to day 7 post plating and allowed to recover. During the recovery phase 4T07 cells were grown in control or fibroblast-conditioned media. Data are of 2 biological replicates. All calculated *P*-values were done using a two-tailed unpaired Student’s *t-*test. Data are the mean +/− s.e.m.

Our data thus far has demonstrated that PDGFR expression increases in response to pemigatinib; however, an exogenous ligand source is needed to stimulate the MAPK pathway and aid in cancer cell survival during drug treatment (Fig. 4). To identify potential ligand sources that may be aiding in the survival of tumor cells during in vivo pemigatinib treatment, we first examined systemic levels of platelet-derived growth factor-AA (PDGF-AA) in blood plasma. We observed a dose-dependent increase in PDGF-AA upon treatment with pemigatinib (Fig. 6a). On a more paracrine level, we postulated that pulmonary tumor fibroblasts could serve as a cell population to support survival of tumor cells during FGFR blockade through their secretion of PDGF ligands [13]. To evaluate this, we isolated pulmonary fibroblasts from tumor naïve BALB/c mice and subjected them to a dose-response of pemigatinib. Ex vivo viability of these fibroblasts was not affected by pemigatinib treatment, and neither was their expression of fibroblast markers, alpha-smooth muscle actin (α-SMA), PDGFR, and Vimentin (Fig. 6b, c). In contrast, real-time polymerase chain reaction (RT-PCR) and enzyme-linked immunosorbent assay (ELISA) analyses suggested an increase in PDGF expression and secretion upon pemigatinib treatment (Fig. 6d, e).

To evaluate the functional impact of lung fibroblasts in supporting tumor cell survival during pemigatinib treatment, we again utilized our 3D culture response and recurrence system. 4T07 cells and primary lung fibroblast cells were cocultured in extracellular matrix, and pemigatinib was used to induce experimental MRD. After pemigatinib treatment, tumor cell recurrence was longitudinally tracked by luciferin-induced bioluminescence (Fig. 6f). Under coculture conditions with pulmonary fibroblasts, 4T07 cells recurred significantly faster following pemigatinib-induced MRD as compared to 4T07 cells alone (Fig. 6f). Consistent with this effect being mediated by secreted factors, similar results could be obtained through supplementation with fibroblast-conditioned media (Fig. 6g). These findings clearly indicate that pulmonary fibroblasts contribute to tumor cell survival and recovery from FGFR-targeted therapy.

### Depletion of PDGFR delays tumor relapse following pemigatinib

**Fig. 7:**
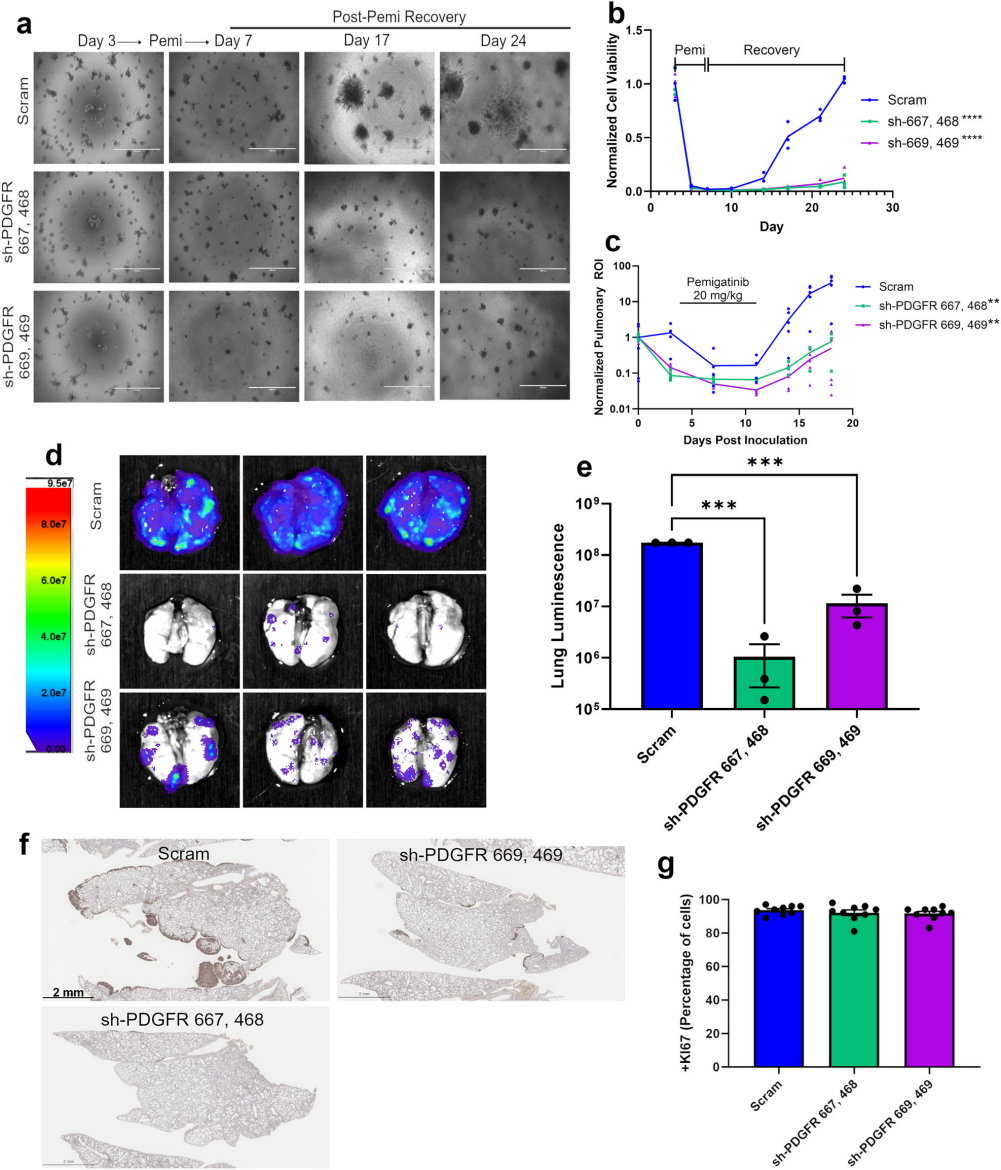
Depletion of PDGFR delays tumor relapse following pemigatinib. **a** Control and PDGFR-depleted 4T07 cells were grown under 3D culture conditions and treated with pemigatinib from days 3–7 post-plating and cells were allowed to recover. Brightfield images were taken at the indicated days. **b** Cell viability was normalized to day 3 and longitudinally tracked over time by bioluminescence. Data are 3 biological replicates. The *P*-value was calculated using a two-way ANOVA. **c** Control and PDGFR-depleted 4T07 cells were inoculated into the tail vein of 6-week-old BALB/c female mice (*n* = 5 per group). Pemigatinib was dosed to all groups on days 4–11 at 20 mg/kg, po, qd. Normalized pulmonary BLI ROI was tracked over time. The *P*-value was calculated using a two-way ANOVA. **d** On day 18 post–tumor cell engraftment, mice were necropsied and ex vivo lung BLI were obtained. **e** Quantified total pulmonary BLI from the 3 biological replicates shown in panel d. **f** Lungs from tumor-bearing mice were processed for histological sectioning and stained for Ki67. **g** The percentage of Ki67+ cells within tumor nodules were counted for each of the three groups. Data represent quantified fields derived from 3 tumor nodules for each of the 3 biological replicates. Calculated *P*-values were done using a two-tailed unpaired Student’s *t-*test. Data are the mean +/− s.e.m.

To further investigate the role of PDGFR in facilitating tumor recurrence following pemigatinib treatment, we depleted both PDGFRα and PDGFRβ in the 4T07 cells (Supplementary Fig. 3a). Again, using our 3D culture response and recurrence assay, we were able to quantify a significant decrease in tumor cell recovery following pemigatinib-induced experimental MRD (Fig. 7a, b). Depletion of PDGFR did not alter pulmonary tumor growth of the 4T07 cells under control conditions or upon treatment with a lower dose of pemigatinib that only causes tumor stasis (10 mg/kg) (Supplementary Fig. 3b–c). However, treatment with a more effective dose of pemigatinib (20 mg/kg) caused a deeper MRD in cells lacking PDGFR, leading to a significant delay in recurrence (Fig. 7c). One week after pemigatinib dosing ended, mice were euthanized and ex vivo BLI was used to visualize pulmonary tumor recurrence (Fig. 7d). Quantification of BLI from the ex vivo lungs demonstrated a significant decrease in recurrence by both knockdown cell lines (Fig. 7e). Histological analyses showed that the tumor nodules that were present had equal frequency of Ki67 staining (Fig. 7f, g; Supplementary Fig. 3e). Taken together, these data indicate that PDGFR expression promotes tumor cellular persistence during periods of FGFR inhibition, but its expression is not involved in tumor cell proliferation following cessation of therapy.

### DNA methyltransferase inhibition delays recurrence following pemigatinib-induced MRD

**Fig. 8:**
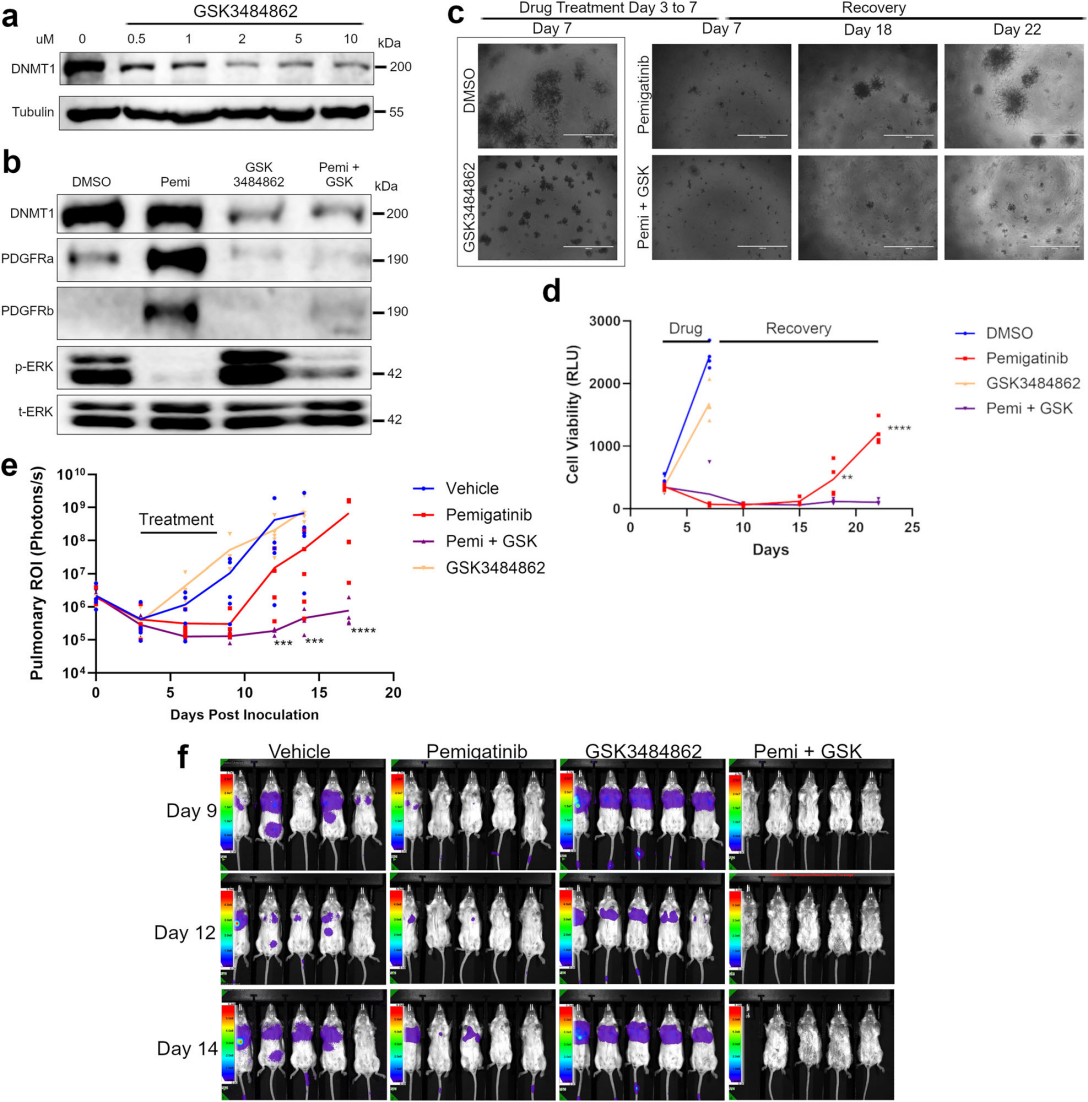
DNMT1 inhibition enhances anti-tumor effects of pemigatinib. **a** 4T07 cells were treated with the DNMT1 inhibitor, GSK3484862, for 72 h at the indicated concentrations. Cells were lysed and analyzed by immunoblot for DNMT1 and tubulin. **b** 4T07 cells were treated with pemigatinib (10 nM) or GSK3484862 (2 µM) for 72 h. Cells were lysed and immunoblotted for DNMT1, PDGFR (α and β), and ERK1/2 (phospho and total). **c** 4T07 cells were grown under 3D culture conditions for 3 days. Cells were subsequently treated with pemigatinib (50 nM), GSK3484862 (1 µM), or a combination of both compounds from days 3 to 7. Following this treatment cells were allowed to recover in the absence of drug. Brightfield images of the 3D cultures were taken at the indicated time points post plating. **d** Cell viability of 4T07 3D cultures were quantified over time using bioluminescence. Data are three biological replicates. Calculated *P*-values were done using a two-tailed unpaired Student’s *t-*test. Data are the mean +/− s.e.m. **e** 4T07 cells (5 × 10^5^) were inoculated into the lateral tail vein of female BALB/c mice. Three days post inoculation mice randomized prior to (*n* = 5 per group) control, pemigatinib (20 mg/kg, po, qd for 3 days then 10 mg/kg, po, qd for additional 2 days), GSK3484862 (50 mg/kg, po, bid) or a combination of both compounds. Calculated *P*-values were done using a two-tailed unpaired Student’s *t-*test. Data are the mean +/− s.e.m. Pulmonary ROI from BLIs were taken at the indicated time points. **f** Representative BLI from days each group at the indicated time points.

As opposed to direct targeting of PDGFR enzymatic activity through application of additional kinase inhibitors or use of less selective compounds, we sought to limit core processes of cellular plasticity that occur through epigenetic rewiring [14–16]. Previous studies demonstrate that DNA methylation contributes to the expression of PDGFR through reorganization of its topological-associated domain, allowing for access of its enhancer to the proximal promoter [17]. Application of an effective dose of the cytosine analogue pan-DNA methyltransferase (DNMT) inhibitor 5-azacytidine was able to partially suppress the ability of pemigatinib to induce PDGFR protein expression (Supplementary Fig. 4a–b). More specifically, application of GSK3484862, a selective enzymatic inhibitor of DNMT1, also potently suppressed the ability of pemigatinib to induce PDGFR expression (Fig. 8a, b, Supplementary Fig. 4c).

To explore therapeutic application of this dual treatment, we utilized our 3D culture and in vivo pemigatinib response and recurrence systems. Established 3D cultures of the 4T07 cells were treated with pemigatinib and GSK3484862 alone or in combination from days 3 to 7, post-plating. Pemigatinib treatment induced experimental MRD irrespective of GSK3484862, whereas GSK3484862 alone had no effect on cell growth (Fig. 8c, d). However, upon cession of treatment we observed that the addition of GSK3484862 prevented recurrence following pemigatinib-induced experimental MRD (Fig. 8c, d). We next established a tolerable yet effective in vivo dose for GSK3484862 by assessing depletion of DNMT1 (Supplementary Fig. 4). Analogous to our in vitro data, GSK3484862 as a monotherapy did not alter pulmonary tumor growth following tail vein inoculation of the 4T07 cells (Fig. 8e). As previously observed, pemigatinib regressed pulmonary tumors to MRD, but these cells aggressively recurred upon cessation of therapy (Fig. 8e). In contrast, combined treatment of pemigatinib and GSK3484862 significantly delayed pulmonary tumor recurrence as compared to pemigatinib treatment alone (Fig. 8e, f).

## Discussion

Overcoming resistance to targeted therapies is necessary to improve cancer patient outcomes. MBC possesses a high degree of cellular plasticity that contributes to cellular persistence upon therapeutic pressure and tumor recurrence [18, 19]. Herein, we were able to develop a unique and efficient in vivo model of advanced disease regression, MRD, and recurrence using tail vein delivery of tumor cells, BLI, and pemigatinib treatment. This system allowed us to discover an acquired mechanism of cellular persistence through upregulation of PDGFR. Inhibition of FGFR with pemigatinib drove robust upregulation of cell surface PDGFRα and PDGFRβ. These data suggest that in the absence of FGFR signaling, BC can convert to PDGFR signaling to drive its survival. Supporting this conclusion, analysis of matched MBC patient samples before and while on treatment with an FGFR inhibitor also demonstrated increased PDGFR expression. The corollary to this supposition was also supported by our data showing FGF stimulation can reduce PDGFR. Additionally, PDGFR expression was also reduced in patients bearing constitutively activating mutations of FGFR.

Functionally, only once PDGFR upregulation occurs under the pressure of pemigatinib are cells able to respond to PDGF. Supplementation of exogenous PDGF ligand aided in the persistence of cells upon treatment with pemigatinib. Flow cytometry and time course experiments demonstrated that pemigatinib does not select for a preexisting subclonal population of PDGFR-expressing cells, but in fact an active upregulation of PDGFR occurs. As a result, a bypass-acquired mechanism of resistance emerges due to growth factor receptor switching. However, once the therapeutic pressure of pemigatinib is withdrawn, FGFR signaling is restored and cells lose PDGFR expression, further illustrating the plasticity of these cells. Tumor relapse was delayed but not prevented upon depletion of PDGFR. This suggests that dual targeting of FGFR/PDGFR would be insufficient for complete eradication of MRD because other mechanisms of persistence would likely emerge.

Given that expression of PDGFR is associated with DNA methylation, we targeted this epigenetic process as a means to limit cellular plasticity [17]. The intricate mechanisms by which FGFR signaling regulates PDGFR remains to be definitively defined. Addition of the DNMT1 inhibitor GSK3484862 did seem to enhance Erk1/2 phosphorylation that was independent of pemigatinib. However, we reasoned that restoration of CTCF binding around the PDGFR locus would insulate the enhancer from the proximal promoter, and dominate over any mechanism by which pemigatinib induces PDGFR expression. Along these lines, the dual inhibition of DNMT1 and FGFR prevented PDGFR upregulation and led to reduced cellular persistence as compared to FGFR inhibition alone.

We identified pulmonary-derived fibroblasts as a source of PDGF ligand that supports cellular persistence in response to pemigatinib. Isolation of primary lung fibroblasts showed that this population was not sensitive to pemigatinib cytotoxicity despite their expression of FGFR1. These data suggest that previous findings that FGFR blockade can break down immune barriers established by cancer-associated fibroblasts in primary tumors may not be applicable to MBCs in the pulmonary environment [20]. These types of differences in drug response by organ-specific fibroblast populations further complicate therapeutic targeting and illustrate the importance of understanding specific primary and metastatic tumor immune microenvironments.

Consistent with our data here, previous findings from our lab and others as well as clinical data indicate that FGFR signaling is associated with immune exclusion [10, 20–23]. Inhibition of FGFR enhances tumor-infiltrating lymphocytes and limits the presence of MDSCs [10, 21, 22, 24, 25]. Due to these strong connections between FGFR targeting and immune modulation, combination of FGFR inhibitors with immune therapy will likely be clinically advantageous [26]. Recent clinical reports are beginning to optimize this therapeutic approach [26].

Overall, the studies presented here developed a unique model of MRD to demonstrate a mechanism of cellular persistence following tumor regression induced by specific inhibition of a receptor tyrosine kinase. Our findings clearly illustrate the ability of cells to alter growth factor signaling profiles to survive in an MRD state, and then revert to their original growth program to induce tumor relapse. This MRD state of persistence is supported by pulmonary fibroblasts through secretion of growth factors that are enhanced upon drug pressure. An epigenetic approach of inhibiting DNMT activity limited cellular plasticity and delayed tumor recurrence. Overall, these mechanistic data support the broader concept of using epigenetic modifiers in conjunction with targeted therapies to reduce persistence and prolong response times.

## Methods

### Cell lines and cell culture

The cell lines used in this study have been grown by the growth conditions as outlined in Supplementary Table S1. The 4T07, D2.OR, and D2.A1 cell lines were obtained from the Fred Miller Laboratory of Wayne State University, Detroit, MI, USA. Knockdown constructs were generated using lentiviral particles with targeted sequences listed in Supplementary Table S2. All of cell lines have been verified through IDEXXIMBAT III CellCheck (IDEXX). All cell lines are routinely PCR-confirmed to be free of Mycoplasmic contamination.

### Primary mouse lung fibroblast isolation

A tumor-naïve BALB/c mouse was euthanized and the whole lung was aseptically dissected out and homogenized in phosphate-buffered serum (PBS). Homogenized lung was incubated in 1 mg/mL collagenase at 37 °C for 30 min with gentle agitation. The cell suspension was centrifuged for 5 min at 1000 g at room temperature. The supernatant was carefully decanted and discarded. The pellet was washed with PBS, and then the cells were pelleted again. A second enzymatic digestion was completed in 0.25% trypsin EDTA and incubated at 37 °C for 20 min. Cells were pelleted by centrifugation and washed with PBS. The cell pellet was resuspended in red blood cell lysis buffer to lyse remaining blood cells. It was wash with PBS. The cell pellet was resuspended in DMEM supplemented with 10% of fetal bovine serum (FBS), 1% pen/strep and place into a tissue culture dish. Cells were cultured at 37 °C and 5% CO_2_. The media was changed every other day until the plate reached confluency. All experiments were completed on the lung fibroblasts within a maximum of 3 passages.

### Animal care, dosing

Pulmonary tumors were induced by injecting 4T07 cells (5 × 10^5^) into the lateral tail vein of 4–6-week-old BALB/c female mice, which were purchased from Jackson Laboratories. Pemigatinib was purchased from MedChemExpress^®^ and administered QD via oral gavage at the indicated concentration with a vehicle composition of 0.5% carboxymethylcellulose and 10% dimethylacetamide (DMAC). The dose volume was administered at 10 mL/kg. GSK3484862 (MedChemExpress^®^) was formulated with 10% DMAC and 90% PEG400 and dosed at a volume of 5 mL/kg BID via oral gavage. At the end of the study, the mice were sacrificed, and primary tumors or tumor-bearing lung(s) were fixed (10% formaldehyde) for 24–48 h. Tissue sectioning (paraffin), H&E staining (Hematoxylin/Eosin/H&E), and IHC were performed by HistoWiz, Inc. Pulmonary tumor growth was monitored after intraperitoneal Luciferin (GoldBio) injection via AMI HT (Spectral Instruments). Mice were randomized before dose initiation according to their pulmonary region of interest (ROI) luminescence values. Completion of the mouse study from Fig. 8 was done in alternate housing conditions. The housing room temperature was elevated and remained at approximately 27 °C for the duration of the study.

### Pulmonary tumor isolation/digestion and flow cytometry

Following the euthanasia of the mice at the study endpoint, the tumor-bearing lungs were extracted and dissociated with the assistance of the Miltenyi tumor dissociation kit and the GentleMACS Disassociator. Whole blood was collected via cardiac puncture and stored in K3 EDTA tubes. Subsequently, 100 µL of blood was used for flow cytometry. The single-cell suspension was then filtered through 70 μm sterile cell strainers and subjected to ACK buffer to lyse the red blood cells. The single-cell suspensions were incubated with TruStain fcX at a 1:50 concentration and Zombie violet at a 1:100 concentration. The pulmonary tumor single-cell suspension was divided into two sections and stained with lymphoid and myeloid antibody panels at a 1:200 dilution. The whole blood was only subjected to the myeloid panel. The antibodies are listed in Supplementary Table S3. The stained cells were then fixed with 4% formaldehyde. Flow cytometry was conducted within 1 week of the staining. The results were analyzed in a closed-label approach with FlowJo software (FlowJo (10.0)).

### Immunoblotting

To perform immunoblot assays, cell lysates were prepared by lysing the samples with modified RIPA lysis buffer containing 50 mol/L Tris; 150 mol/L NaCl; 0.25% Sodium deoxycholate; 1% NP40; 0.1% SDS; Protease Inhibitor Cocktail (Sigma); 10 mol/L Sodium orthovanadate; 40 mmol/L B-glycerol phosphate; and 20 mmol/L Sodium fluoride. After SDS PAGE and Transfer, the PVDF membrane was probed with antibodies listed in Supplementary Table S3.

### RNA isolation and quantitative real-time PCR analysis

RNA lysates were prepared using the Invitrogen™ PureLink™ RNA Mini Kit per manufacturer instructions. RNA from fresh frozen tumor biopsy specimens was extracted using the miRNeasy Kit (Qiagen) per the manufacturer’s protocol. Synthesis of cDNA was completed using Applied Biosystems™ High-Capacity cDNA Reverse Transcription Kit followed by RT-PCR using Thermo Scientific™ Maxima SYBR Green/ROX qPCR Master Mix. Primers used can be found in Supplementary Table S5.

### Cell viability of lung fibroblasts

To evaluate the cell viability of primary lung fibroblasts after treatment, the Promega CellTiter-Glo^®^ reagent was used according to the manufacturer’s instructions. Data was normalized to a DMSO treatment condition.

### ELISA

The ELISA used to detect PDGF-AA was purchased from Thermo Scientific (REF: EM61RB). Both conditioned media and blood plasma were tested for PDGF-AA using this kit according to the manufacturer’s instructions. Concentration calculation used a 4-parameter logistic curve fit based off the standards to find concentrations of the unknowns.

### In vitro 3D growth assays

To grow cells in 3D culture, 50 µL of Bio-Teche^®^ R&D system’s cultrex RGF (REF: 3433-001-01) was put into each well of a 96-well plate. After at least 30 min incubation at 37 °C, 2,000 cells were seeded onto the 3D matrix in media that contained 5% cultrex in total volume of 200 µL. To evaluate the cell viability of each well, 2 µL of luciferin was added to each well and after 30 min incubation at room temperature, the luminescence of each well was read on a Promega GloMax plate reader.

### Statistical analysis

GraphPad Prism 10 was used for all statistical analysis. All data met the assumptions of the test and test were consider justified and appropriate. In vitro assay data differences between two groups were compared using Student’s *t-*test. The standard error of the mean (s.e.m.) is indicated by error bars. A two-way analysis of variance (ANOVA) test was used to compare the measurements for in vivo experiments. Sample sizes were estimated based on known variance of the approaches. Statistical significance was reflected beginning with a *p-*value of <0.05. No exclusion criteria were used.

### Ethics approval and consent to participate

All in vivo studies were conducted according to and approved by the Purdue University Institutional Animal Care and Use Committee (IACUC). The patient was consented to institutional review board –approved study OSU-13053, *Precision Cancer Medicine for Advanced Cancer through High-throughput Sequencing* (The Ohio State University, Columbus, OH). This study (NCT02090530) allows for serial evaluation of blood and tumor specimens for genomic analyses and additional ongoing cancer research.

## Supplementary information

Supplemental Tables

Supplemental Figure 1

Supplemental Figure 2

Supplemental Figure 3

Supplemental Figure 4

Supplementary Figure Legends

Original Data

## Data Availability

All data generated or analyzed during this study are included in this published article (and its supplementary information files.
