# Supplementary material for: Growth factor receptor plasticity drives therapeutic persistence of metastatic breast cancer
Source: Cell Death Dis. 2025 Apr 4;16(1):251. doi: 10.1038/s41419-025-07591-3 (PMC11971261; doi:10.1038/s41419-025-07591-3)
Supplement: Supplementary file 1 — Supplemental Tables [file 41419_2025_7591_MOESM1_ESM.docx]

**Supplementary Table 1 Cell lines and culture conditions**

| Name of the cell line | Culture condition |
| --- | --- |
| D2.A1 | DMEM with 10% Fetal Bovine Serum (FBS) |
| 4TO7 | DMEM with 10% FBS |
| D2.OR | DMEM with 10% FBS |
| Mouse Lung Fibroblast Primary Cells | DMEM with 10% FBS |

**Supplementary Table 2 Targeting sequences of shRNA vectors**

| Simplified ID | Source Clone ID | Target | Vector | Targeting Sequence |
| --- | --- | --- | --- | --- |
| 667 | TRCN0000055003 | Pdgfra Mouse | pLKO.1 PuroR | TAGAGGGTAATAAGAGCTGGC |
| 669 | TRCN0000055005 | Pdgfra Mouse | pLKO.1 PuroR | TTTGTTTCTCACTTCTCCAGG |
| 468 | VB900139-2468nan | Pdgfrb Mouse | pLKO.1 Hygro | GATGATCTGCAAGCATATTAA |
| 469 | VB900139-2469nte | Pdgfrb Mouse | pLKO.1 Hygro | AGTGGCCATTACACCATTATA |

**Supplementary Table 3 Primary and secondary antibodies used in this study**

| Antibody | Source | Identifier | Host |
| --- | --- | --- | --- |
| PE anti-mouse CD45 | BioLegend | Catalog No: #103105 | Rat |
| PE/Cyanine7 anti-mouse/human CD11b | BioLegend | Catalog No: #101215 | Rat |
| FITC anti-mouse Ly-6G | BioLegend | Catalog No: #127605 | Rat |
| APC anti-mouse Ly-6C | BioLegend | Catalog No: #128015 | Rat |
| FITC anti-mouse CD45 | BioLegend | Catalog No: #157213 | Rat |
| PE anti-mouse CD8a | BioLegend | Catalog No: #162303 | Rat |
| Brilliant Violet 711™ anti-mouse CD4 | BioLegend | Catalog No: #100549 | Rat |
| PE/Dazzle™ 594 anti-mouse CD279 (PD-1) | BioLegend | Catalog No: #135227 | Rat |
| APC anti-mouse CD140b | BioLegend | Catalog No: #136007 | Rat |
| PE anti-mouse CD140a | BioLegend | Catalog No: #135905 | Rat |
| Phospho-p44/42 MAPK (Erk1/2) (Thr202/Tyr204) Antibody | Cell Signaling Technology | Catalog No: #9101 | Rabbit |
| p44/42 MAPK (Erk1/2) Antibody | Cell Signaling Technology | Catalog No: #9102 | Rabbit |
| FGF Receptor 1 (D8E4) XP^®^ Rabbit mAb | Cell Signaling Technology | Catalog No: #9740 | Rabbit |
| α-Smooth Muscle Actin | Cell Signaling Technology | Catalog No: #19245 | Rabbit |
| Vimentin | Cell Signaling Technology | Catalog No: #5741 | Rabbit |
| PDGF Receptor α (D1E1E) XP^®^ Rabbit mAb | Cell Signaling Technology | Catalog No: #3174 | Rabbit |
| PDGF Receptor β (28E1) Rabbit mAb | Cell Signaling Technology | Catalog No: #3169 | Rabbit |
| Tubulin, beta | Developmental Studies Hybridoma Bank (DSHB) | Catalog No: E7 | Mouse |
| Goat anti-Mouse IgG (H+L) Secondary Antibody, HRP | ThermoFisher | Catalog No: 62-6520 | Goat |
| Goat anti-Rabbit IgG (H+L) Secondary Antibody, HRP | ThermoFisher | Catalog No: 65-6120 | Goat |

**Supplementary Table 4 Growth factors and inhibitors**

| Growth factors / Inhibitors/Antibody | Source | Identifier |
| --- | --- | --- |
| Pemigatinib (INCB054828) | MedChemExpress | HY-109099 |
| Futibatinib | MedKoo Biosciences, Inc. | 206535 |
| GSK-3484862 | MedChemExpress | HY-135146 |
| 5-Azacytidine | MedChemExpress | HY-10586 |
| Anti-PD-1 (RMP1-14) | ichorbio | ICH1132 |
| Rat IgG2a In Vivo Isotype Control | ichorbio | ICH2244 |
| InVivoMAb anti-mouse PD-L1 (B7-H1) Clone: 10F.9G2™ | BioXCell | BE0101 |
| InVivoMAb rat IgG2b isotype control  Clone: LTF-2 | BioXCell | BE0090 |
| Basic FGF (FGF2), Human | GoldBio | Catalog No: 1140-02-10 |
| Recombinant Human PDGF-BB Protein, CF | R&D systems | Catalog No: 220-BB-010 |

**Supplementary Table 5 qPCR primers**

| Gene Target | Forward | Reverse |
| --- | --- | --- |
| PDGFRα (mouse) | GACTTCCTAAAGAGTGACCATCC | CTTCCCAGTCCTTCAGCTTATC |
| PDGFRβ (mouse) | AGGACAACCGTACCTTGGGTGACT | CAGTTCTGACACGTACCGGGTCTC |
| GAPDH (mouse) | CAACTTTGGCATTGTGGAAGGGCTC | GCAGGGATGATGTTCTGGGCAGC |
| PDGF-a (mouse) | GTACTGAATTTCGCCGCCAC | GTATCTCGGCTTCCTCGGC |
| PDGF-b (mouse) | CCTGCAGTGAACTTTGGAGC | CTCAGCCCCATCTTCATCTACG |
|  |  |  |
