# Supplementary figures and images for: Growth factor receptor plasticity drives therapeutic persistence of metastatic breast cancer
Source: Cell Death Dis. 2025 Apr 4;16(1):251. doi: 10.1038/s41419-025-07591-3 (PMC11971261; doi:10.1038/s41419-025-07591-3)

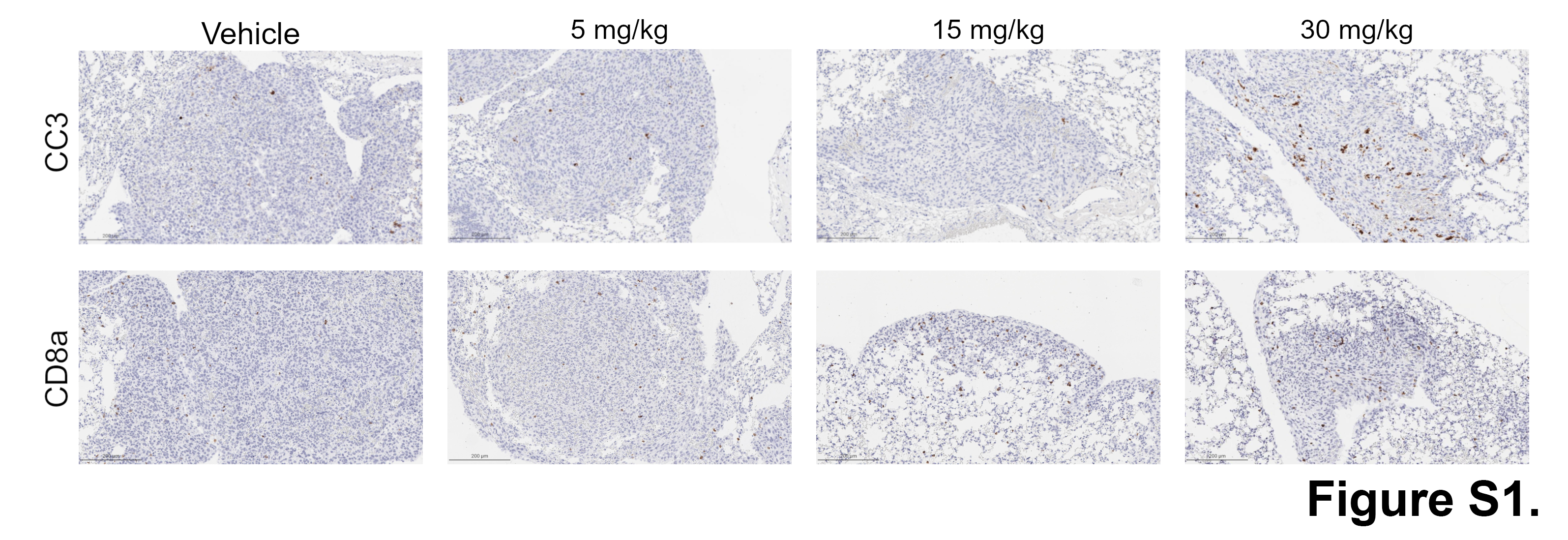

Supplement: Supplementary file 2 — Supplemental Figure 1 [file 41419_2025_7591_MOESM2_ESM.png]

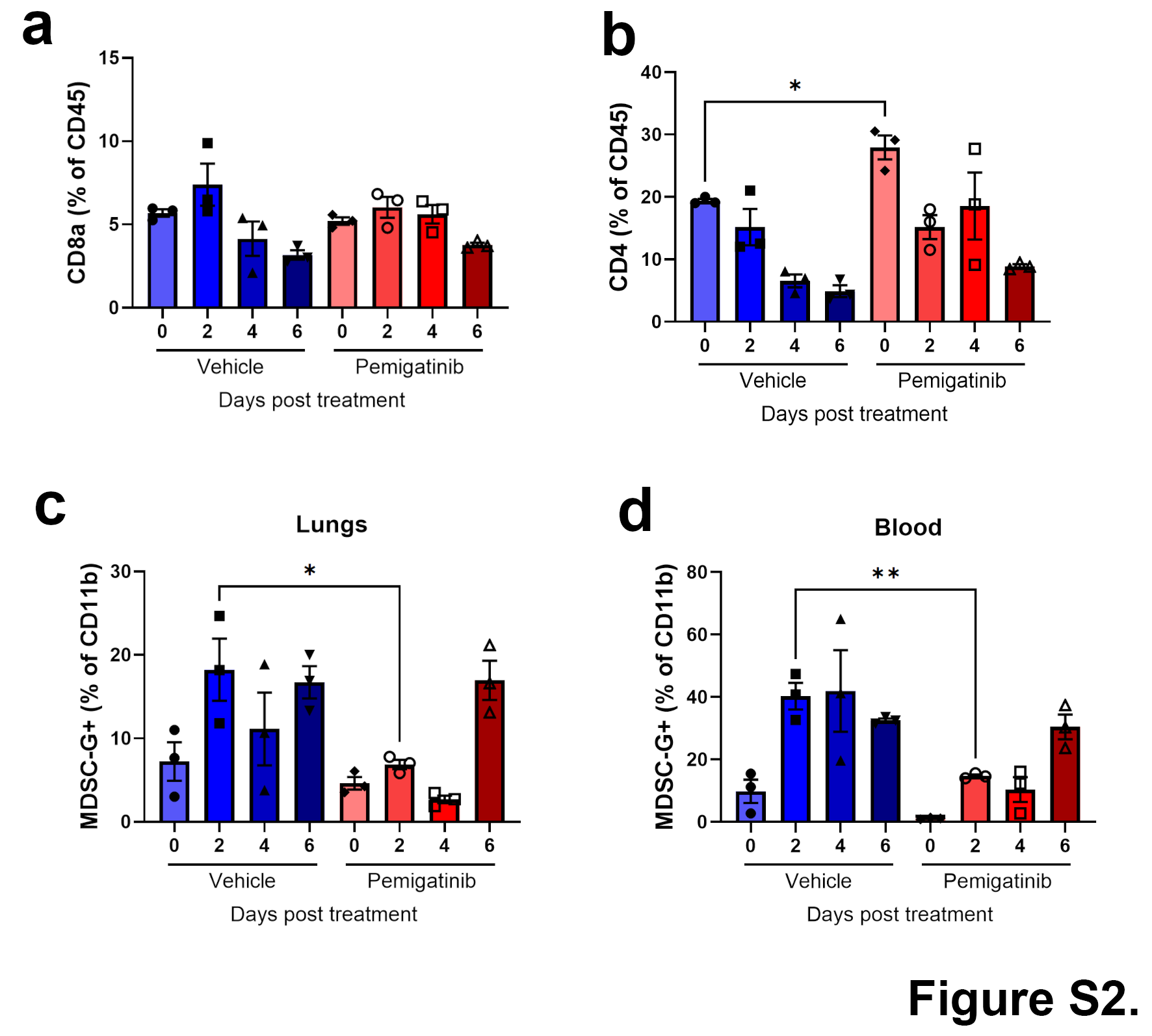

Supplement: Supplementary file 3 — Supplemental Figure 2 [file 41419_2025_7591_MOESM3_ESM.png]

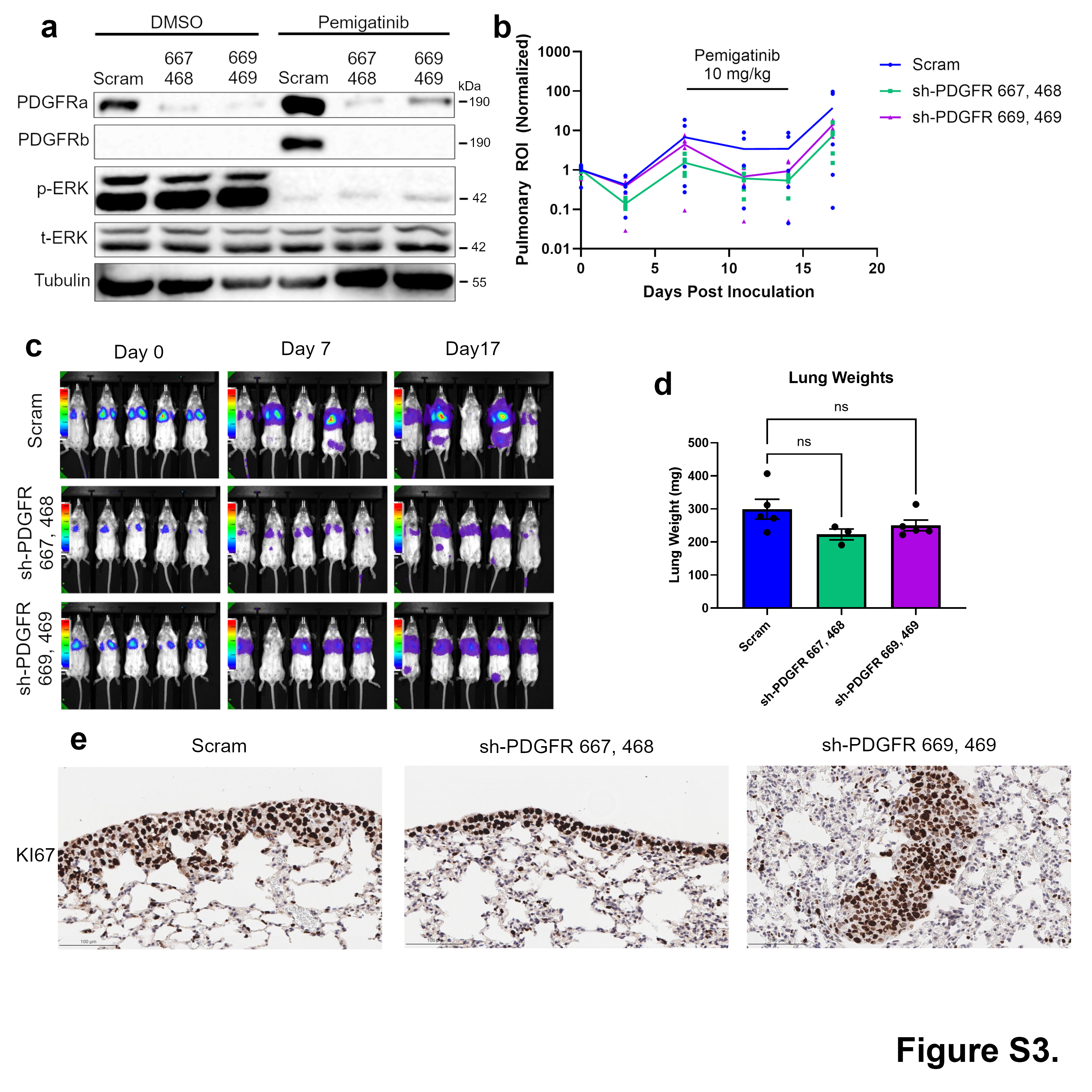

Supplement: Supplementary file 4 — Supplemental Figure 3 [file 41419_2025_7591_MOESM4_ESM.png]

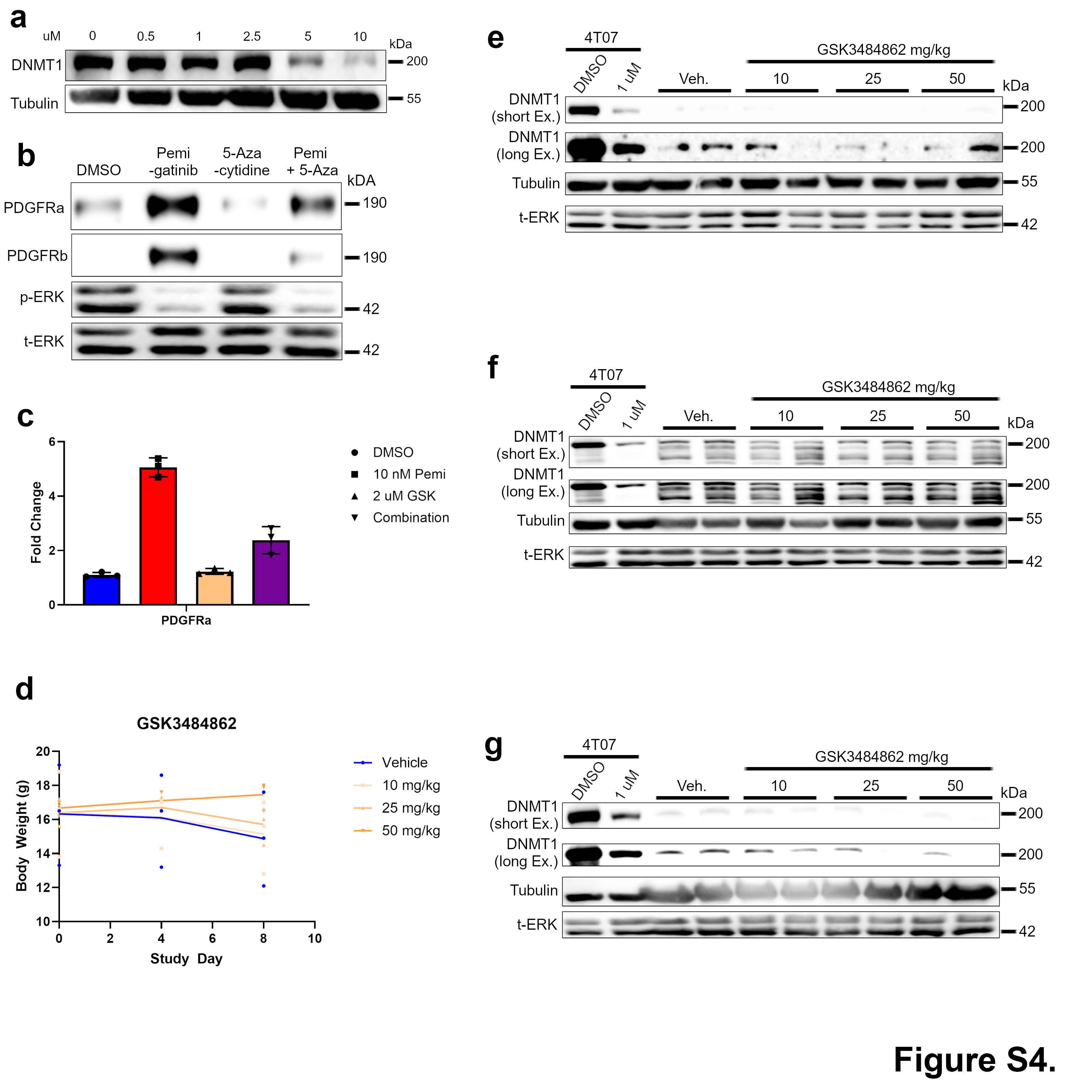

Supplement: Supplementary file 5 — Supplemental Figure 4 [file 41419_2025_7591_MOESM5_ESM.png]
