# Supplementary material for: Growth factor receptor plasticity drives therapeutic persistence of metastatic breast cancer
Source: Cell Death Dis. 2025 Apr 4;16(1):251. doi: 10.1038/s41419-025-07591-3 (PMC11971261; doi:10.1038/s41419-025-07591-3)
Supplement: Supplementary file 6 — Supplementary Figure Legends [file 41419_2025_7591_MOESM6_ESM.docx]

**Supplementary Figure Legends**

**Supplementary Fig. 1**

IHC images from the experiments described in Figure 1. Sections were stained for cleaved caspase 3 (CC3) and CD8a.

**Supplementary Fig. 2**

**a–d** Flow cytometric analyses from the experiments described in Figure 5b. Cells were isolated from lungs and prepared as single cell suspensions. **a** Lung CD8a+ as a percentage of CD45+ flow cytometry. **b** Lung CD4+ as a percentage of CD45+ flow cytometry. **c** Lung G-MDSC (Ly6C–, Ly6G+) as a percentage of cd11b+ flow cytometry. **d** Whole blood G-MDSC (Ly6C–, Ly6G+) as a percentage of cd11b+ flow cytometry. Data represents 3 biological replicates. All calculated *P*-values were done using a two-tailed unpaired Student’s *t-*test. Data are the mean +/- s.e.m.

**Supplementary Fig. 3**

**a** Stable lentiviral knockdown constructs sh-PDGFRa (667 or 669) and sh-PDGFRb (468 or 469) were established and verified by western blot in untreated and pemigatinib-treated conditions. Immunoblot was completed for PDGFR (α and β), ERK1/2 (phospho and total), and tubulin. **b** Female BALB/c mice (n = 5) were inoculated with 4T07 control cells expressing a scrambled (scram) shRNA, or shRNA targeting PDGFRa and b (sh-PDGFR 667 468 or sh-PDGFR 669 469) at 5 × 10^5^ cells per mouse. Starting on day 7 mice began treatment with 10 mg/kg pemigatinib. The graph represents the pulmonary ROI values from BLI during the experiment. **c** BLI from days 0, 7, and 17. **d–e** *In vivo* experiment from Fig. 7c after necropsy lungs were weighed and processed for IHC. **e** Ki67 staining from Fig 7g.

**Supplementary Fig. 4**

**a** 4T07 cells were treated with the DNMT inhibitor, 5-azacytidine, for 72 hours at the indicated micromolar concentration then blotted for DNMT1 and tubulin. **b** 4T07 cells were treated with pemigatinib (10 nM) or 5-azacytidine (5 µM) for 72 hours then blotted for DNMT1, PDGFR (α and β), and ERK1/2 (phospho and total). **c** RT-PCR was completed after a 72-hour treatment of pemigatinib (10 nM) or GSK3484862 (2 µM) on 4T07 cells. Data represents the mean of 3 biological replicates +/- the s.e.m. **d** *In vivo* treatment of tumor-naive female BALB/c mice with GSK3484862 at 10, 25, or 50 mg/kg po bid from day 0 to 8. The graph represents body weights throughout the treatment. **e** After dosing with GSK3484862, tissue was isolated from mice and prepared for immunoblot blot analyses alongside *in vitro* GSK3484862-treated 4T07 cells. Immunoblots are two biological replicates of *in vivo-*treated mice. Lysate were derived from **e** lungs, **f** spleen, **g** and brain.
